# Use of C-reactive protein to guide the antibiotic therapy in hospitalized patients: a systematic review and meta-analysis

**DOI:** 10.1186/s12879-023-08255-3

**Published:** 2023-05-03

**Authors:** Raphael Figuiredo Dias, Ana Clara Rivetti Bitencourt de Paula, Ursula Gramiscelli Hasparyk, Marcos de Oliveira Rabelo Bassalo Coutinho, João Rafael Assis Alderete, Júlia Chihondo Kanjongo, Renata Aguiar Menezes Silva, Nathalia Sernizon Guimarães, Ana Cristina Simões e Silva, Vandack Nobre

**Affiliations:** 1 Laboratório Interdisciplinar de Investigação Médica (LIIM), Universidade Federal de Minas Gerais (UFMG), Belo Horizonte, MG Brazil; 2 School of Medicine, Universidade Federal de Minas Gerais (UFMG), Belo Horizonte, MG Brazil; 3 School of Medicine, Faculdade de Saúde E Ecologia Humana (FASEH), Vespasiano, MG Brazil; 4 Núcleo Interdisciplinar de Investigação Em Medicina Intensiva (NIIMI), Universidade Federal de Minas Gerais (UFMG), Belo Horizonte, MG Brazil; 5 Internal Medicine Department, School of Medicine, Universidade Federal de Minas Gerais (UFMG), Belo Horizonte, MG Brazil

**Keywords:** Adult, C-reactive protein (CRP), Anti-bacterial agents, Duration of therapy, Circulating biomarkers

## Abstract

**Background:**

C-reactive protein (CRP) is an inflammatory protein used in clinical practice to identify and monitor inflammatory and infectious processes. Recent data suggest CRP might be useful in guiding antibiotic therapy discontinuation among critical care patients. This meta-analysis analyzed the benefits and risks of CRP-guided protocols to guide antibiotic therapy in hospitalized patients in comparison with standard treatment.

**Methods:**

Studies were searched in four databases: CENTRAL, Medline, Embase and LILACS. The search was performed until Jan 25th, 2023. The reference lists of the articles retrieved and related review studies were hand-screened to find eligible trials that might have been missed. Primary endpoints included the duration of antibiotic therapy for the index episode of infection. The secondary endpoint was the all-cause hospital mortality and infection relapses. The risk of bias was evaluated using the Cochrane Risk of Bias 2.0 tool. Random effects were used to pool the mean differences and odds ratio of individual studies. The protocol was registered in PROSPERO (CRD42021259977).

**Results:**

The search strategy retrieved 5209 titles, out of which three studies met the eligibility criteria and were included in this meta-analysis. 727 adult patients were analyzed, of whom 278 were included in the intervention group and 449 were included in the control group. 55,7% of all patients were women. Meta-analysis indicated that experimental groups (CRP-guided) had a lower duration of antibiotic therapy (days) [MMD = -1.82, 95%IC -3.23; -0.40]; with no difference in mortality [OR = 1.19 95%IC 0.67–2.12] or in the occurrence of infection relapse [OR = 3.21 95%IC 0.85–12.05].

**Conclusion:**

The use of CRP-guided protocol reduces the total amount of time required for antibiotic therapy when compared to standard protocols of treatment in hospitalized patients with acute bacterial infection. We did not observe statistical differences regarding mortality and infection relapse rates.

**Supplementary Information:**

The online version contains supplementary material available at 10.1186/s12879-023-08255-3.

## Introduction

C-reactive protein (CRP) is a well-known biomarker classically associated with acute inflammatory processes, such as infections, trauma, surgery, tissue necrosis, cell injury, and autoimmune conditions [1–4]. Indeed, the serum concentration of CRP notably rises after the onset of inflammation, mainly in response to IL-6 production, which activates the CRP gene, allowing its expression by hepatocytes. IL-1 and endogenous steroids, to a lesser extent, also contribute to CRP production [2].

CRP has been widely used in clinical practice as an index of ongoing infectious processes during hospitalizations and a marker of the effectiveness of antimicrobial therapy. It has been used in clinical practice for three major roles, with variable levels of evidence: (1) diagnosis support; (2) definition of prognosis, follow-up, and treatment guidance during infectious processes; and (3) screening tool for occult infectious or inflammatory diseases [5]. Recently, CRP has been tested by several studies to help the decision regarding antibiotic discontinuation [6–9], especially in light of the ascending rates of bacterial multi-resistance secondary to the overuse of antibiotics. The comparison between CRP and procalcitonin (PCT) in sepsis recognition and management of antimicrobial therapy is frequently made, however, none of them was consensually recognized as an ideal biomarker for sepsis [10, 11].

We still lack solid evidence to support the use of CRP-based protocols to guide antibiotic therapy duration in hospitalized patients. Hence, in this meta-analysis, we sought to investigate the usefulness and safety of CRP-guided protocols to discontinue antibiotic therapy in hospitalized patients with suspected or confirmed bacterial infection.

## Methods

This systematic review was based on recommendations from the Cochrane Guidelines for Systematic Reviews of Interventions (Cochrane Library, 2021) [12] and was written according to Preferred Reporting Items for Systematic Reviews and Meta-Analyses (PRISMA) guidelines [13]. The review protocol was registered at the PROSPERO under registration CRD42021259977.

During our review process, we made two key changes in our study protocol, as compared to the original PROSPERO registration. The first change refers to the study eligibility criteria of the study. Initially, our purpose was to include only critically-ill adults (18 years old or older) admitted to an ICU environment. However, due to the scarcity of studies in this setting, we opted to include patients hospitalized in the wards or admitted to the emergency room (ER). In addition, we also updated our exclusion criteria to avoid including studies that did not measure our studies' outcomes. The second change was made in the outcomes section. We opted to exclude "length of ICU stay and "length of hospital stay" from our primary outcomes, as well as "free days from antibiotics" from our secondary outcomes since these data were not available in all retrieved studies. Given that only a few studies fulfilled our eligibility criteria, the absence of this data would preclude a reliable comparison among them.

### Search strategy

To identify randomized clinical trials assessing the use of CRP-based protocols to guide the duration of antibiotic therapy, we conducted a comprehensive systematic search using the electronic databases Medline (by PubMed), Embase, CENTRAL (by Cochrane Library), and LILACS (by Biblioteca Virtual em Saúde). Additionally, the articles selected by the systematic search had their references manually reviewed to find eligible trials that might have been missed.

Records were not excluded based on language or date of publication. The search for information was conducted until January 25th, 2023. Descriptors were identified in Medical Subject Headings (MeSH), Descritores em Ciências da Saúde (Decs) and Embase Subject Headings (Emtree). The search strategy was adapted based on descriptors in each database and is presented in the Supplementary Material.

### Outcomes

The primary outcome was the duration of antibiotic therapy for the first episode of infection, in days. Secondary outcomes included: (1) all-cause hospital mortality, and (2) relapse of infection, defined according to the source article's criteria.

### Eligibility criteria

We included randomized controlled trials that evaluated hospitalized adults (18 years old or older) with a clinical indication for antibiotic therapy defined by the assistant medical team. The eligibility criteria involved studies conducted with patients admitted to the intensive care unit (ICU), ward, or emergency room (ER), with suspected or confirmed bacterial infection and in use of antibacterial treatment. We included studies whose protocol compared a CRP-based strategy of antibiotic therapy (intervention) *versus* standard criteria (e.g., local protocols, international guidelines) without CRP or with another biomarker.

We excluded studies that involved patients with bacterial infection requiring long-duration antibiotic therapy (i.e., infective endocarditis, deep pyogenic abscess, osteomyelitis) or severely immunocompromised patients (HIV infection with CD4 + lymphocytes counts < 200 cells/mm^3^ or solid organ or bone marrow transplantation, current intensive antineoplastic chemotherapy and other similar modalities of immunosuppression) and studies not reporting the data required for the measurement of this review's pre-defined endpoints.

Duplicate studies or studies with unclear information—and which we did not receive any response from the corresponding author(s) after email, studies conducted in patients not under treatment for bacterial infections, research that did not evaluate CRP or observational studies, narrative, integrative, systematic reviews, or meta-analysis were excluded. Also, studies that have a non-standard protocol design for CRP evaluation in antibiotic therapy discontinuation were excluded.

### Study selection

Electronic search results from pre-defined databases were uploaded using the Rayyan Qatar Computing Research Institute [14]. After excluding duplicate articles, two authors independently carried out the process of title and abstract exclusion, and a third resolved eventual disagreements. Then, the full text of the remaining articles was checked to evaluate their eligibility.

### Data extraction

Two independent authors extracted information from the selected primary studies and an additional reviewer resolved disagreements. The following information was extracted: author, year of publication, journal name, location, age median, sexes, number of patients (intervention group and control group), CRP-guided protocol (cut-offs, percentage of reduction), CRP test and method, comparator, type of infection, ICU and hospital length of stay, duration of antibiotic use, death, recurrence of infection.

### Risk of bias assessment

Two investigators independently assessed the risk of bias in the selected studies according to the Cochrane Collaboration’s tool for assessing the risk of bias (RoB 2 instrument provided by the Cochrane Collaboration) [15]. Any disagreement was solved by a third reviewer. The responses to the questionnaires could be classified as: “yes”, “no”, “unclear” or “not applicable”. Based on the recommendations of this tool, a judgment of each domain was recorded as “high”, “moderate”, “low” or “very low” risk of bias.

The potential of publication bias was assessed and included as a funnel plot and can be found in supplementary material #2. The quality of evidence assessment was made using GRADE from Cochrane group and is described in Table 1.

**Table 1:** GRADE (Grading of Recommendations, Assessment, Development and Evaluations) assessment. Question: "Should C-reactive protein (CRP) be used to guide the duration of antimicrobial treatment compared to antimicrobial treatment according to best practices in antibacterial use?"

			**Certainty assessment**				**Number of patients**		**Effects**		**Certainty**	**Relevance**
**Number of studies**	**Study design**	**Risk of bias**	**Inconsistencies**	**Indirect evidence**	**Imprecision**	**Other considerations**	**Use of CRP to guide antimicrobial treatment duration**	**Antimicrobial treatment according to best practices in antibacterial use**	**Relative (95% CI)**	**Absolute (95% CI)**		
**Recurrence of infection**												
3	Randomized controlled trials	Not severe	Not severe	Not severe	Not severe	All potential confounding factors reduced the demonstrated effect	9/278 (3.2%)	3/284 (1.1%)	**OR 3.21** (0.86 to 12.05)	**23 more per 1.000 **(from 1 minus to 103 plus)	⨁⨁⨁⨁ High	IMPORTANT
**Mortality**												
3	Randomized controlled trials	Not severe	Not severe	Not severe	Not severe	All potential confounding factors reduced the demonstrated effect	33/278 (11.9%)	30/284 (10.6%)	**OR 1.19** (0.67 to 2.12)	**18 more per 1.000** (from 32 minus to 95 plus)		
**Duration of antibiotic therapy**												
3	Randomized controlled trials	Not severe	Not severe	Not severe	Not severe	All potential confounding factors reduced the demonstrated effect	273	444	-	Mean difference **1.82 days minus** (3.23 minus to 0.4 minus)		

*CI* Confidence interval, OR Odds ratio

### Meta-analysis

A random-effects model was used for pooling the results of included studies, as clinical heterogeneity was expected. The treatment effect was projected by forest plots. Heterogeneity between studies was assessed through Cochran’s Q test, and the p-value for trend < 0.10 was considered statistically significant. The I^2^ test was carried out to evaluate the magnitude of heterogeneity between studies. It was considered low when I^2^ < 25.0%; moderate when I^2^ ≥ 25 and ≤ 75% and high when I^2^ > 75.0%. Analyses were performed in the Review Manager software, version 5.4.

## Results

**Fig. 1:**
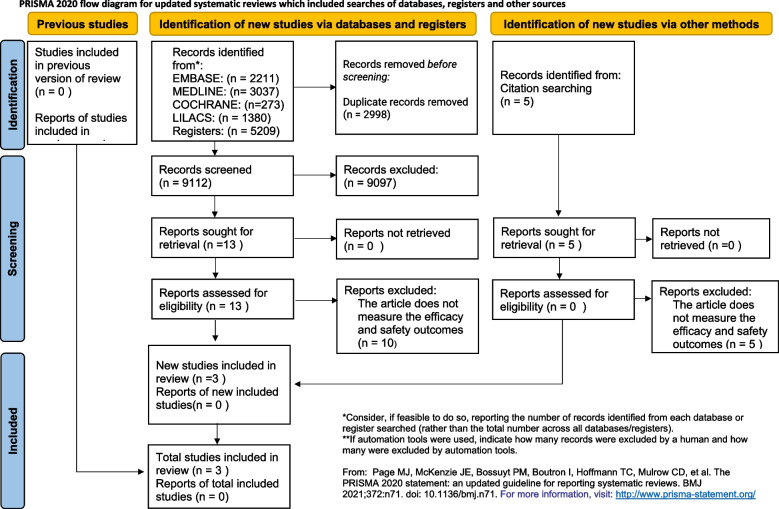
Flow diagram for updated systematic reviews which included searches of databases, registers and other sources according to the Preferred Reporting Items for Systematic Reviews and Meta-Analyses statement guidelines (PRISMA)

The search strategy retrieved 2,196 titles after duplicate records removal, out of which three studies met the eligibility criteria and were included in this meta-analysis (Fig. 1). Assessed studies investigated the value of CRP-based protocols in comparison with non-CRP-based protocols in the task of reducing antibiotic exposure in patients admitted to the ICU, ward, and emergency room. The two studies involving patients admitted to the ward or the emergency room met the pre-specified eligibility criteria for this review. Of the three randomized controlled trials included in the final analysis, two were carried out in two Brazilian university hospitals in 2013 and 2020, respectively, and the last one was carried out in three Switzerland hospitals in 2020. The main characteristics and findings of the included studies are presented in Tables 2 and 3.

**Table 2:** Main characteristics of the included studies

Author, year, journal	General scenario profile	Age and sex distribution	CRP test and method and comparator group
Oliveira, C. F. et al., 2013, Crit Care Med Oct;41(10):2336–2343	Patients with severe sepsis or septic shock, admitted to the intensive care units at two university hospitals in Brazil; the authors compared the duration of antibiotic therapy between the group treated with the aid of a CRP-based protocol versus a procalcitonin-based protocol. A control group without biomarkers was lacking. They observed the PCT protocol was not superior to the CRP protocol level for reducing the use of antibiotics (Duration of antibiotic therapy in PCT protocol: 8.1 (3.7) days vs Duration of antibiotic therapy in CRP protocol: 7.2 (3.5) days; *p* = 0.25)	**Mean age** (years) CRP: 59.6 ± 18.5 Procalcitonin: 59.6 ± 13.3 Total: 59.8 ± 16.8 Male n(%): CRP: 26 (45,61%) Procalcitonin: 31 (54,39%) Total: 57 (60.6%) Females: n(%) CRP: 19 (42,2%) Procalcitonin: 18(48,8%) Total: 37 (39.4%)	Method: Discontinuation of antibiotic therapy based on serum levels of CRP (CRP < 25 mg/L or decrease ≥ 50%) and PCT (PCT < 0.1 ng/ml or decrease ≥ 90%) associated with SOFA score analysis
Borges, I. et al., 2020, Crit Care. Jun;24: 281	Critically-ill patients admitted to the ICU of a university hospital in Brazil; authors compared the duration of antibiotic therapy using a CRP-based protocol *versus* an evidence-based strategy without the use of biomarkers. They observed that the CRP-based strategy was viable in reducing antibiotics exposure (Intervention group median: 7 (5–8.8), Control group median: 7 (7–11.3), *p* = 0.011)	**Median age (years)** CRP: 62 (53–68) Control: 60 (49–70) years Total: 61 (51–68) years Male n(%): CRP: 34 (50%) Control: 34 (50%) Total: 68 (52,3%) Females n(%): CRP: 30 (48,4) Control: 32 (51,6%) Total: 62 (47.7%)	Method: Duration of antibiotic therapy based on serum levels of CRP (CRP < 35 mg/L or decrease ≥ 50%) v*ersus* a standard protocol without the use of biomarkers
von Dach, E. et al., 2020, JAMA Jun;323(21):2160–2169	Patients with gram-negative uncomplicated bacteremia in three Switzerland hospitals; authors compared the duration of antibiotic therapy guided by CRP levels *versus* two fixed-length antibiotic therapy groups (7 and 14 days). It was found that the CRP protocol was noninferior to the fixed-day treatment protocols treatment	**Median age (years)** CRP guided: 78 (69–86) 7 days: 78 (69–86) 14 days: 80 (67–85) Total: 79 (68–86) Male; n(%): CRP guided: 64 (32,5%) 7 days: 62 (31,5) 14 days: 71 (36%) Total: 197 (39%) Females n(%): CRP guided: 105 (34,3%) 7 days: 107 (35%) 14 days: 94 (30,7%) Total: 306 (61%)	Method: Duration of antibiotic therapy based on serum levels of CRP (decrease ≥ 75% from its peak associated with the absence of fever for the previous 48 h) versus a fixed-length of therapy (7 and 14 days)

**Table 3:** Main findings of the included studies

Author, year, journal	Intervention evaluated	Number of intervention patients	CRP cut-off	CRP test and method	Comparator
Oliveira, C. F. et al., 2013, Crit Care Med	CRP-guided discontinuation of antibiotic therapy in patients with sepsis or septic shock. *Patients with a SOFA score > 10 and a positive blood culture result received at least 7 days of antibiotic therapy	45	- CRP < 25 mg/L (if initial CRP < 100 mg/L) or 7 days of antibiotic therapy- Decrease ≥ 50% (if initial CRP ≥ 100 mg/L) or 7 days of antibiotic therapy	Reactive test VITROS (Johnson & Johnson Clinical Diagnostics, Inc., Rochester, NY)	Discontinuation of antibiotic therapy guided by procalcitonin serum levels in patients with sepsis or septic shock considering initial clinical status and the SOFA score. **Patients with a SOFA score > 10 and a positive blood culture result received at least 7 days of antibiotic therapy
Borges, I. et al., 2020, Crit Care Med	Duration of antibiotic therapy guided by daily monitoring of CRP serum levels in critically ill infected adult patients	64	- CRP < 35 mg/L (if initial CRP < 100 mg/L)—CRP decrease ≥ 50% (if initial CRP ≥ 100 mg/L)	Test for the quantitative determination of serum CRP concentration (Vitros-Johnson & Johnson, USA)	Best practices for rational use of antibiotics according to the best evidence established in the literature for duration of antibiotic treatment, microbiological data, and estimated previous time regarding the infectious site
von Dach, E. et al., 2020, JAMA	CRP-guided antibiotic treatment duration in adults hospitalized with gram-negative bacteremia	170	Once serum CRP had decreased to 75% from its peak and if fever was absent for at least 48 h. OR At 14-day of treatment (if by day 14 the CRP level had not decreased to 75% of its maximum value, it was no longer used to guide therapy)	CRP was measured via immunoturbidimetry (more details about the method were not provided)	The CRP group was compared to two fixed-length antibiotic therapy groups (7 and 14 days). Local guidelines oriented antibiotics choice, including changes in the route of administration when appropriate

A total of 727 patients were analyzed, of whom 278 were included in the intervention group (CRP) and 449 were included in the control group. Men comprised 44.3% of all patients (322 subjects), whereas 55.7% were women (405 subjects). The average age of the patients included in the three studies was 59.8 ± 16.8 years (mean ± SD) (*Oliveira *et al.,* 2013*), 61 years (51 – 68) (*Borges *et al*.,* 2020), and 79 years (68–86) (*von Dach *et al., 2020).

### Outcomes

**Fig. 2:**
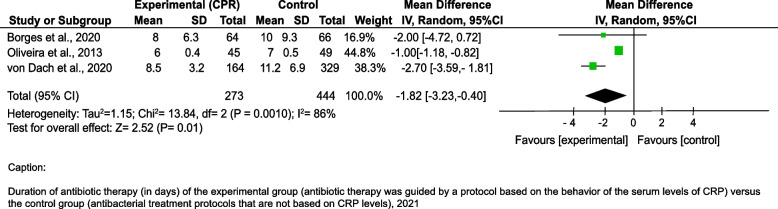
Duration of antibiotic therapy (in days) of the experimental group (antibiotic therapy was guided by a protocol based on the behavior of the serum levels of CRP) versus the control group (antibacterial treatment protocols that are not based on CRP levels), 2021

Regarding the duration of antibiotic therapy (in days), pooled results from the random-effects model indicated that experimental groups (CRP-guided) had a lower duration of antibiotic therapy (days) compared to the control groups [Mean difference = -1.82, 95%IC -3.23; -0.40]. There was significant heterogeneity among the studies I2 = 86% (Fig. 2).

**Fig. 3:**
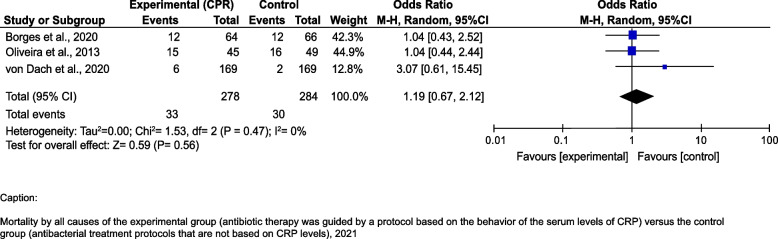
Mortality by all causes of the experimental group (antibiotic therapy was guided by a protocol based on the behavior of the serum levels of CRP) *versus* the control group (antibacterial treatment protocols that are not based on CRP levels), 2021

The pooled results from the random-effects model indicated that no difference in mortality was observed between the intervention groups and the control groups [OR = 1.19 95%IC 0.67–2.12]. There was no heterogeneity among the studies I2 = 0% (Fig. 3).

**Fig. 4:**
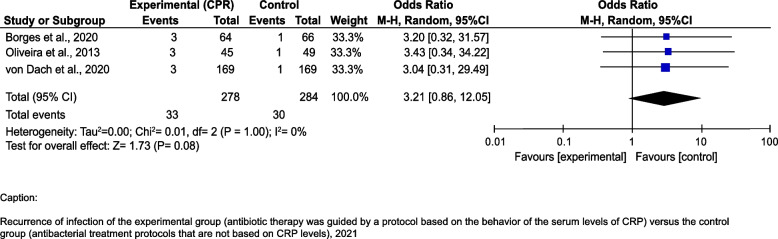
Recurrence of infection of the experimental group (antibiotic therapy was guided by a protocol based on the behavior of the serum levels of CRP) versus the control group (antibacterial treatment protocols that are not based on CRP levels), 2021

Likewise, there was no significant difference in the occurrence of infection relapse between the groups using the CRP-guided strategy and the control groups [OR = 3.21 95%IC 0.85–12.05]. There was no heterogeneity among the studies I2 = 0% (Fig. 4).

Only two of the three studies have measured the length of stay in the hospital and ICU. *Borges *et al. [8] found that the length of stay in the hospital was longer in the CRP group than in the control group (31.5 (16–53) vs 25.5 (15–43)); however, this difference was not statistically significant (p-value 0.356). The same group found no difference between the groups at the length of stay in the ICU (CRP—8 (4–15); Control—8 (4–17); p-value 0.414)). Similarly, *Oliveira *et al. [9] found that the length of stay was not statistically significant in both scenarios, despite the shorter length of stay recorded in CRP groups, both in ICU (CRP—12 (7–18); PCT—14 (9–24); *p*-value 0.164) and hospital (CRP—25 (13–52); 36 (20–59); *p*-value 0.175).

## Discussion

In this systematic review and meta-analysis, we found that the CRP-guided strategy reduced the duration of antibiotic therapy in hospitalized patients with acute bacterial infections without apparent harm. Antibiotic stewardship programs require the implementation of many complementary actions to obtain positive and consistent results. Biomarkers' guidance of antibiotic therapy is one of these strategies, with increasing evidence of benefit during the last two decades. Most of the high-level evidence in this field comes from studies using procalcitonin (PCT) as the guide biomarker, notably in patients with respiratory tract infections. Many original studies and individual data meta-analyses have shown the efficacy and safety of PCT to safely reduce antibiotic exposure, with an apparent improvement in mortality [16].

The widespread use of PCT as a tool to guide antibiotic therapy is limited by the elevated costs of this marker and its poor availability in low and medium-income countries. Therefore, CRP arises as an interesting alternative, since it is a cheaper and widely available biomarker compared to PCT. In addition, clinicians have much more experience with CRP in their daily practice. Observational studies have demonstrated that CRP behaviour during antibiotic therapy is highly associated with mortality among hospitalized patients with severe infections [17]. Thus, patients with a marked decline of CRP levels during the first four to five days of antibiotic therapy have a better outcome as compared to those on which CRP remains elevated [18, 19]. These findings support the hypothesis that CRP can be used to identify candidates for a shorter anti-infectious therapy.

In recent years, some studies have suggested that CRP might be as useful as PCT to help in the strategy of rational use of antibiotics. More specifically, CRP-guided protocols have been tested to guide the decision of antibiotic therapy interruption among hospitalized patients, using well-controlled standard care as comparators. Recently, *Borges *et al*.* [8] showed a reduction of one day in the median duration of antibiotic therapy for the first episode of infection (from 7 to 6 days) among critically ill patients with suspected or confirmed infection. These results were in accordance with the study of *Von Dach *et al*.*, [7] which showed that the CRP-guided strategy and a fixed length of 7 days of antibiotic therapy were not inferior to a fixed length of 14 days of treatment for uncomplicated gram-negative bacteremia.

In a randomized controlled trial to test a protocol guided by CRP concentration as compared to a PCT-based strategy, *Oliveira *et al*.* [9] found that the former approach was not inferior in reducing the length of antibiotic therapy, namely in primo infection cases. It was also observed that a ceiling of seven days of antibiotics is safe for most patients with sepsis, regardless of the support of biomarkers. This finding was corroborated by PCT in other studies with a similar context [9].

Despite allowing a lower antibiotic exposure, CRP protocols used in the intervention groups of the studies included in this review were not associated with a higher mortality rate. In two of the three studies analyzed, the absolute number of deaths was higher in CRP groups, but this finding was not statistically relevant. Concerning infection relapse—an important parameter to identify inefficiency of treatment and clinical failure—no statistical difference was observed between the groups.

The need for judicious use of antibiotics is recognized in the main guidelines of recommendation for sepsis management, even though adding biomarkers such as PCT to the clinical evaluation in the decision of discontinuing antibiotic therapy has not been recognized as a high-evidenced approach [17]. Reasons to explain this interpretation of the literature data are the inevitably open-label nature of the intervention in the published trials, limitations regarding safety issues, and scarcity of studies proving that these biomarkers-based strategies are cost-effective. As mentioned above, due to its large availability, we believe CRP may be a suitable candidate for this goal.

This study has two main limitations that deserve to be mentioned. First, only three studies were eligible for our review and there was heterogeneity among them regarding one of the outcomes of interest. This finding is likely assigned to clinical heterogeneity, reflecting the different characteristics of the infectious condition presented by the patients enrolled in the three studies. Also, we were not able to stratify our analysis according to the site or severity of the infection. However, we believe that the low number of studies available is an additional reason to gather their results aiming to generate more robust evidence. Despite the single-center nature and the small sample of participants included in two of these studies, all of them had a good performance in the methodological quality assessment. Second, two out of the three studies included in this review were conducted by the same research team and all of them were single, double or triple center studies. All these issues certainly limit the generalizability (external validation) of their findings.

The number of trials testing the role of CRP to guide antibiotic therapy is scarce and new studies are desirable. Of note, some important points should be considered in these trials' protocols. The decision to stop antibiotics in patients with a good clinical and biochemical response (ie, fast decrease in CRP levels) seems safe and it is in line with the modern recommendations regarding antibiotic use [20, 21]. However, those with persistently elevated CRP levels despite a full course of antibiotics (ie., for about one week) represent a major challenge. In these cases, it is essential to rule out the presence of occult infectious focus, multiresistant bacteria, non-bacterial etiology or overlapping nosocomial infections. If all these conditions are absent, prolonging antibiotic therapy is probably useless and potentially harmful [22, 23]. A better understanding of the pathogenic mechanisms behind elevated CRP levels in these patients might contribute to a more assertive approach. Finally, strategies of biomarkers combination to guide decisions regarding antibiotic therapy constitute a promising prospect in this field.

## Conclusion

In this meta-analysis of three randomized controlled trials we found that, as compared to standard control groups based on the best current evidence for antibiotic therapy, a CRP-guided strategy safely reduces the length of treatment with antibiotics in hospitalized patients with acute bacterial infections. Large well-designed multicenter studies are highly desirable to confirm our findings.

## Supplementary Information

**Additional file 1.**

**Additional file 2.**

## Data Availability

All data generated or analyzed during this study are included in this published article [and its supplementary information files.

## Abbreviations

CRP: C-reactive protein
Decs: Descritores em Ciências da Saúde (Descriptors in Health Sciences)
Emtree: Embase Subject Headings
ER: Emergency room
ICU: Intensive care unit
IL-1: Interleukin-1
IL-6: Interleukin-6
MeSH: Medical Subject Headings
PCT: Procalcitonin
PRISMA: Preferred Reporting Items for Systematic Reviews and Meta-Analyses
SOFA score: Sequential Organ Failure Assessment score
