# Supplementary material for: Use of C-reactive protein to guide the antibiotic therapy in hospitalized patients: a systematic review and meta-analysis
Source: BMC Infect Dis. 2023 May 3;23:276. doi: 10.1186/s12879-023-08255-3 (PMC10155296; doi:10.1186/s12879-023-08255-3)
Supplement: Supplementary file 1 — Additional file 1. [file 12879_2023_8255_MOESM1_ESM.docx]

**SUPPLEMENTARY MATERIAL #1**

**Use of C-reactive protein to guide the antibiotic therapy in hospitalized patients:**

**a systematic review and meta-analysis**

Raphael Figuiredo Dias*^1,3a^*, Ana Clara Rivetti Bitencourt de Paula*^2,5a^*, Ursula Gramiscelli Hasparyk^1,3^, Marcos de Oliveira Rabelo Bassalo Coutinho^3^, João Rafael Assis Alderete^1^, Júlia Chihondo Kanjongo^3^, Renata Aguiar Menezes Silva^1^, Nathalia Sernizon Guimarães^4b^, Ana Cristina Simões e Silva^1b^, Vandack Nobre^4,5b^

*^a^ Both authors contributed equally to this article as first authors.*

*^b^ All authors contributed equally to this article as senior authors.*

*^1^ Interdisciplinary Laboratory of Medical Investigation (LIIM), School of Medicine, Universidade Federal de Minas Gerais (UFMG), Belo Horizonte, MG, Brazil*

*^2^ School of Medicine, Faculdade de Saúde e Ecologia Humana (FASEH, Vespasiano, MG, Brazil)*

*^3^ School of Medicine, Universidade Federal de Minas Gerais (UFMG), Belo Horizonte, MG, Brazil*

*^4^ Internal Medicine Department, School of Medicine, Universidade Federal de Minas Gerais (UFMG), Belo Horizonte, MG, Brazil*

*^5^ Núcleo Interdisciplinar de Investigação em Medicina Intensiva (NIIMI), Universidade Federal de Minas Gerais (UFMG), Belo Horizonte, MG, Brazil*

**Address correspondence to this author at the Dr. Vandack Nobre, Internal Medicine Department, School of Medicine, Universidade Federal de Minas Gerais (UFMG), Belo Horizonte, MG, Brazil, Avenida Professor Alfredo Balena, 110, Hospital das Clínicas, Unidade de Cuidados Intensivos do Adulto, Terceiro Andar, Ala Leste - Santa Efigênia, ZIP Code: 30130-100 Belo Horizonte, Brazil. E-mail: vandack@gmail.com*

**2023**

# SEARCH STRATEGY

**Are the C-reactive protein (CRP) levels helpful to guide the antibiotic therapy in adult**

**patients admitted to the ward and ICU?**

# PubMed/Medline – 29 papers found.

#1 "Adult"[Mesh] OR "Adult" OR “Adults” OR"young adult"[MeSH Terms] OR "young adult"[MeSH] OR "young adult" OR "middle aged"[MeSH Terms] OR "middle aged" OR "elderly" OR "elderlies" OR "elderly's" OR "elderlys" OR "aged, 80 and over"[MeSH] OR "80 and over aged" OR "oldest" OR "nonagenarian" OR "nonagenarians" OR "centenarian" OR "centenarians"

#2 “Anti-Bacterial Agents” [Mesh] OR “Agents, Anti-Bacterial” OR “Anti Bacterial Agents” OR “Antibacterial Agents” OR “Agents, Antibacterial” OR “Antibacterial Agent” OR “Agent, Antibacterial” OR “Anti-Bacterial Compounds” OR “Anti Bacterial Compounds” OR “Compounds, Anti-Bacterial” OR “Anti-Bacterial Agent” OR “Agent, Anti-Bacterial” OR “Anti Bacterial Agent” OR “Anti-Bacterial Compound” OR “Anti Bacterial Compound” OR “Compound, Anti-Bacterial” OR “Bacteriocidal Agents” OR “Agents, Bacteriocidal” OR “Bacteriocidal Agent” OR “Agent, Bacteriocidal” OR “Bacteriocide” OR “Bacteriocides” OR “Anti-Mycobacterial Agents” OR “Agents, Anti-Mycobacterial” OR “Anti Mycobacterial Agents” OR “Anti-Mycobacterial Agent” OR “Agent, Anti-Mycobacterial” OR “Anti Mycobacterial Agent” OR “Antimycobacterial Agent” OR “Agent, Antimycobacterial” OR “Antimycobacterial Agents” OR “Agents, Antimycobacterial” OR “Antibiotics” OR “Antibiotic”

#3 “C-Reactive Protein” [Mesh] OR “C Reactive Protein” OR “hsCRP” OR “High Sensitivity C-Reactive Protein” OR “High Sensitivity C Reactive Protein” OR “hs-CRP”

#4 “Duration of Therapy” [Mesh] OR “Therapy Duration” OR “Duration of Treatment” OR “Treatment Duration”

#5 ((clinical[Title/Abstract] AND trial[Title/Abstract]) OR clinical trials as topic[MeSH Terms] OR clinical trial[Publication Type] OR random*[Title/Abstract] OR random allocation[MeSH Terms] OR therapeutic use[MeSH Subheading])

**Total: 29 papers**

Search: (((("Adult"[Mesh] OR "Adult" OR "Adults" OR"young adult"[MeSH Terms] OR "young adult"[MeSH] OR "young adult" OR "middle aged"[MeSH Terms] OR "middle aged" OR "elderly" OR "elderlies" OR "elderly's" OR "elderlys" OR "aged, 80 and over"[MeSH] OR "80 and over aged" OR "oldest" OR "nonagenarian" OR "nonagenarians" OR "centenarian" OR "centenarians") AND ("Anti-Bacterial Agents" [Mesh] OR "Agents, Anti-Bacterial" OR "Anti Bacterial Agents" OR "Antibacterial Agents" OR "Agents, Antibacterial" OR "Antibacterial Agent" OR "Agent, Antibacterial" OR "Anti-Bacterial Compounds" OR "Anti Bacterial Compounds" OR "Compounds, Anti-Bacterial" OR "Anti-Bacterial Agent" OR "Agent, Anti-Bacterial" OR "Anti Bacterial Agent" OR "Anti-Bacterial Compound" OR "Anti Bacterial Compound" OR "Compound, Anti-Bacterial" OR "Bacteriocidal Agents" OR "Agents, Bacteriocidal" OR "Bacteriocidal Agent" OR "Agent, Bacteriocidal" OR "Bacteriocide" OR "Bacteriocides" OR "Anti-Mycobacterial Agents" OR "Agents, Anti-Mycobacterial" OR "Anti Mycobacterial Agents" OR "Anti-Mycobacterial Agent" OR "Agent, Anti-Mycobacterial" OR "Anti Mycobacterial Agent" OR "Antimycobacterial Agent" OR "Agent, Antimycobacterial" OR "Antimycobacterial Agents" OR "Agents, Antimycobacterial" OR "Antibiotics" OR "Antibiotic")) AND (Duration of Therapy [Mesh] OR "Therapy Duration" OR "Duration of Treatment" OR "Treatment Duration")) AND (((clinical[Title/Abstract] AND trial[Title/Abstract]) OR clinical trials as topic[MeSH Terms] OR clinical trial[Publication Type] OR random*[Title/Abstract] OR random allocation[MeSH Terms] OR therapeutic use[MeSH Subheading]))) AND ("C-Reactive Protein" [Mesh] OR "C Reactive Protein" OR "hsCRP" OR "High Sensitivity C-Reactive Protein" OR "High Sensitivity C Reactive Protein" OR "hs-CRP")

("Adult"[MeSH Terms] OR "Adult"[All Fields] OR "Adults"[All Fields] OR "young adult"[MeSH Terms] OR "young adult"[MeSH Terms] OR "young adult"[All Fields] OR "middle aged"[MeSH Terms] OR "middle aged"[All Fields] OR "elderly"[All Fields] OR "elderlies"[All Fields] OR "elderly's"[All Fields] OR "elderlys"[All Fields] OR "aged, 80 and over"[MeSH Terms] OR "80 and over aged"[All Fields] OR "oldest"[All Fields] OR "nonagenarian"[All Fields] OR "nonagenarians"[All Fields] OR "centenarian"[All Fields] OR "centenarians"[All Fields]) AND ("anti bacterial agents"[MeSH Terms] OR "agents anti bacterial"[All Fields] OR "anti bacterial agents"[All Fields] OR "Antibacterial Agents"[All Fields] OR "agents antibacterial"[All Fields] OR "Antibacterial Agent"[All Fields] OR "agent antibacterial"[All Fields] OR "anti bacterial compounds"[All Fields] OR "anti bacterial compounds"[All Fields] OR ("anti bacterial agents"[Pharmacological Action] OR "anti bacterial agents"[MeSH Terms] OR ("anti bacterial"[All Fields] AND "agents"[All Fields]) OR "anti bacterial agents"[All Fields] OR ("compounds"[All Fields] AND "anti"[All Fields] AND "bacterial"[All Fields])) OR "anti bacterial agent"[All Fields] OR ("anti bacterial agents"[Pharmacological Action] OR "anti bacterial agents"[MeSH Terms] OR ("anti bacterial"[All Fields] AND "agents"[All Fields]) OR "anti bacterial agents"[All Fields] OR ("agent"[All Fields] AND "anti"[All Fields] AND "bacterial"[All Fields])) OR "anti bacterial agent"[All Fields] OR "anti bacterial compound"[All Fields] OR "anti bacterial compound"[All Fields] OR ("anti bacterial agents"[Pharmacological Action] OR "anti bacterial agents"[MeSH Terms] OR ("anti bacterial"[All Fields] AND "agents"[All Fields]) OR "anti bacterial agents"[All Fields] OR ("compound"[All Fields] AND "anti"[All Fields] AND "bacterial"[All Fields])) OR "Bacteriocidal Agents"[All Fields] OR ("anti bacterial agents"[Pharmacological Action] OR "anti bacterial agents"[MeSH Terms] OR ("anti bacterial"[All Fields] AND "agents"[All Fields]) OR "anti bacterial agents"[All Fields] OR ("agents"[All Fields] AND "bacteriocidal"[All Fields])) OR "Bacteriocidal Agent"[All Fields] OR ("anti bacterial agents"[Pharmacological Action] OR "anti bacterial agents"[MeSH Terms] OR ("anti bacterial"[All Fields] AND "agents"[All Fields]) OR "anti bacterial agents"[All Fields] OR ("agent"[All Fields] AND "bacteriocidal"[All Fields])) OR "Bacteriocide"[All Fields] OR "Bacteriocides"[All Fields] OR "anti mycobacterial agents"[All Fields] OR ("anti bacterial agents"[Pharmacological Action] OR "anti bacterial agents"[MeSH Terms] OR ("anti bacterial"[All Fields] AND "agents"[All Fields]) OR "anti bacterial agents"[All Fields] OR ("agents"[All Fields] AND "anti"[All Fields] AND "mycobacterial"[All Fields])) OR "anti mycobacterial agents"[All Fields] OR "anti mycobacterial agent"[All Fields] OR "agent anti mycobacterial"[All Fields] OR "anti mycobacterial agent"[All Fields] OR "Antimycobacterial Agent"[All Fields] OR ("anti bacterial agents"[Pharmacological Action] OR "anti bacterial agents"[MeSH Terms] OR ("anti bacterial"[All Fields] AND "agents"[All Fields]) OR "anti bacterial agents"[All Fields] OR ("agent"[All Fields] AND "antimycobacterial"[All Fields])) OR "Antimycobacterial Agents"[All Fields] OR "agents antimycobacterial"[All Fields] OR "Antibiotics"[All Fields] OR "Antibiotic"[All Fields]) AND ("duration of therapy"[MeSH Terms] OR "Therapy Duration"[All Fields] OR "Duration of Treatment"[All Fields] OR "Treatment Duration"[All Fields]) AND (("clinical"[Title/Abstract] AND "trial"[Title/Abstract]) OR "clinical trials as topic"[MeSH Terms] OR "clinical trial"[Publication Type] OR "random*"[Title/Abstract] OR "random allocation"[MeSH Terms] OR "therapeutic use"[MeSH Subheading]) AND ("c reactive protein"[MeSH Terms] OR "c reactive protein"[All Fields] OR "hsCRP"[All Fields] OR "high sensitivity c reactive protein"[All Fields] OR "high sensitivity c reactive protein"[All Fields] OR "hs-CRP"[All Fields])

Translations

Duration of Therapy [Mesh]: "duration of therapy"[MeSH Terms]

clinical trials as topic[MeSH Terms]: "clinical trials as topic"[MeSH Terms]

random allocation[MeSH Terms]: "random allocation"[MeSH Terms]

therapeutic use[MeSH Subheading]: "therapeutic use"[Subheading]

Updated (25 Jan 2023): 7 results added

# Embase- 96 papers found.

#1 ('adult'/syn OR 'aged/syn OR ´middle age'/syn OR 'young adult'/syn) AND [embase]/lim

#2 (‘antibiotic agent' OR ‘antimycobacterial agent’ OR ‘antispirochetal agent’) AND [embase]/lim

#3 ('C-Reactive Protein '/syn) AND [embase]/lim

#4 ('Treatment duration'/syn) AND [embase]/lim

#5 'clinical trial'/de OR 'randomized controlled trial'/de OR 'randomization'/de OR 'single blind procedure'/de OR 'double blind procedure'/de OR 'crossover procedure'/de OR 'placebo'/de OR 'prospective study'/de OR ('randomi?ed controlled' NEXT/1 trial*) OR rct OR 'randomly allocated' OR 'allocated randomly' OR 'random allocation' OR (allocated NEAR/2 random) OR (single NEXT/1 blind*) OR (double NEXT/1 blind*) OR ((treble OR triple) NEAR/1 blind*) OR placebo*

('adult'/syn OR 'aged/syn or ´middle age' OR 'young adult'/syn) AND ('antibiotic agent' OR 'antimycobacterial agent' OR 'antispirochetal agent') AND 'c-reactive protein'/syn AND 'treatment duration'/syn AND [embase]/lim AND ('clinical trial'/de OR 'randomized controlled trial'/de OR 'randomization'/de OR 'single blind procedure'/de OR 'double blind procedure'/de OR 'crossover procedure'/de OR 'placebo'/de OR 'prospective study'/de OR ('randomi?ed controlled' NEXT/1 trial*) OR rct OR 'randomly allocated' OR 'allocated randomly' OR 'random allocation' OR (allocated NEAR/2 random) OR (single NEXT/1 blind*) OR (double NEXT/1 blind*) OR ((treble OR triple) NEAR/1 blind*) OR placebo)

Updated (25 Jan 2023): 25 results added

# Web of Science / CINAHL - 0 papers found.

"Adult" OR "Adult" OR "Adults" OR "young adult" OR "young adult" OR "young adult" OR "middle aged" OR "middle aged" OR "elderly" OR "elderlies" OR "elderly's" OR "elderlys" OR "aged, 80 and over" OR "80 and over aged" OR "oldest" OR "nonagenarian" OR "nonagenarians" OR "centenarian" OR "centenarians"

"Anti-Bacterial Agents" OR "Agents, Anti-Bacterial" OR "Anti Bacterial Agents" OR "Antibacterial Agents" OR "Agents, Antibacterial" OR "Antibacterial Agent" OR "Agent, Antibacterial" OR "Anti-Bacterial Compounds" OR "Anti Bacterial Compounds" OR "Compounds, Anti-Bacterial" OR "Anti-Bacterial Agent" OR "Agent, Anti-Bacterial" OR "Anti Bacterial Agent" OR "Anti-Bacterial Compound" OR "Anti Bacterial Compound" OR "Compound, Anti-Bacterial" OR "Bacteriocidal Agents" OR "Agents, Bacteriocidal" OR "Bacteriocidal Agent" OR "Agent, Bacteriocidal" OR "Bacteriocide" OR "Bacteriocides" OR "Anti-Mycobacterial Agents" OR "Agents, Anti-Mycobacterial" OR "Anti Mycobacterial Agents" OR "Anti-Mycobacterial Agent" OR "Agent, Anti-Mycobacterial" OR "Anti Mycobacterial Agent" OR "Antimycobacterial Agent" OR "Agent, Antimycobacterial" OR "Antimycobacterial Agents" OR "Agents, Antimycobacterial" OR "Antibiotics" OR "Antibiotic"

“Duration of Therapy” OR "Therapy Duration" OR "Duration of Treatment" OR "Treatment Duration"

clinical AND trial OR clinical trials as topic OR clinical trial OR random*OR random allocation OR therapeutic use

"C-Reactive Protein" OR "C Reactive Protein" OR "hsCRP" OR "High Sensitivity C-Reactive Protein" OR "High Sensitivity C Reactive Protein" OR "hs-CRP"

Updated (25 Jan 2023): 0 results added

#

# Central - 37 papers found.

37 Trials matching "Adult" OR "Adult" OR "Adults" OR "young adult" OR "young adult" OR "young adult" OR "middle aged" OR "middle aged" OR "elderly" OR "elderlies" OR "elderly's" OR "elderlys" OR "aged, 80 and over" OR "80 and over aged" OR "oldest" OR "nonagenarian" OR "nonagenarians" OR "centenarian" OR "centenarians" in Title Abstract Keyword AND "Anti-Bacterial Agents" OR "Agents, Anti-Bacterial" OR "Anti Bacterial Agents" OR "Antibacterial Agents" OR "Agents, Antibacterial" OR "Antibacterial Agent" OR "Agent, Antibacterial" OR "Anti-Bacterial Compounds" OR "Anti Bacterial Compounds" OR "Compounds, Anti-Bacterial" OR "Anti-Bacterial Agent" OR "Agent, Anti-Bacterial" OR "Anti Bacterial Agent" OR "Anti-Bacterial Compound" OR "Anti Bacterial Compound" OR "Compound, Anti-Bacterial" OR "Bacteriocidal Agents" OR "Agents, Bacteriocidal" OR "Bacteriocidal Agent" OR "Agent, Bacteriocidal" OR "Bacteriocide" OR "Bacteriocides" OR "Anti-Mycobacterial Agents" OR "Agents, Anti-Mycobacterial" OR "Anti Mycobacterial Agents" OR "Anti-Mycobacterial Agent" OR "Agent, Anti-Mycobacterial" OR "Anti Mycobacterial Agent" OR "Antimycobacterial Agent" OR "Agent, Antimycobacterial" OR "Antimycobacterial Agents" OR "Agents, Antimycobacterial" OR "Antibiotics" OR "Antibiotic" in Title Abstract Keyword AND “Duration of Therapy” OR "Therapy Duration" OR "Duration of Treatment" OR "Treatment Duration" in Title Abstract Keyword AND clinical AND trial OR clinical trials as topic OR clinical trial OR random*OR random allocation OR therapeutic use in Title Abstract Keyword AND "C-Reactive Protein" OR "C Reactive Protein" OR "hsCRP" OR "High Sensitivity C-Reactive Protein" OR "High Sensitivity C Reactive Protein" OR "hs-CRP" in Title Abstract Keyword - (Word variations have been searched)

Updated (25 Jan 2023): 0 results added

1. **LILACS - 0 papers found.**

#1 "Adulto" OR "Adult" OR "Adulto Jovem" OR "Adultos Jovens" OR "Jovem Adulto" OR "Young Adult" OR "Adulto Joven" OR "Idoso" OR "Idosos" OR "Pessoa de Idade" OR "Pessoa Idosa" OR "Pessoas de Idade" OR "Pessoas Idosas" OR" População Idosa" OR" Aged" OR "Anciano"

#2 "Antibacterianos" OR "Agente Antimicobacteriano" OR "Agentes Antibacterianos" OR "Agentes Antimicobacterianos" OR "Agentes Bactericidas" OR "Antibacteriano" OR "Antibiótico" OR "Antibióticos" OR "Antimicobacteriano" OR "Antimicobacterianos" OR "Bactericida" OR "Bactericidas" OR "Compostos Antibacterianos" OR "Fármaco Antimicobacteriano" OR "Fármacos Antibacterianos" OR "Fármacos Antibióticos" OR "Fármacos Antimicobacterianos" OR "Germicida" OR "Germicidas" OR "Medicamentos Antibióticos" OR "Anti-Bacterial Agents" OR "Antibacterianos"

#3 "Proteína C-Reativa" OR "C-Reactive Protein"

#4 "Duração da Terapia" OR "Duração do Tratamento" OR "Duration of Therapy" OR "Duración de la Terapia"

Updated (25 Jan 2023): 0 results added
