# Supplementary material for: Use of C-reactive protein to guide the antibiotic therapy in hospitalized patients: a systematic review and meta-analysis
Source: BMC Infect Dis. 2023 May 3;23:276. doi: 10.1186/s12879-023-08255-3 (PMC10155296; doi:10.1186/s12879-023-08255-3)
Supplement: Supplementary file 2 — Additional file 2. [file 12879_2023_8255_MOESM2_ESM.docx]

**SUPPLEMENTARY MATERIAL #2**

**Use of C-reactive protein to guide the antibiotic therapy in hospitalized patients: a systematic review and meta-analysis**

Raphael Figuiredo Dias*^1a^* , Ana Clara Rivetti Bitencourt de Paula*^2,5a^*, Ursula Gramiscelli Hasparyk^3^, Marcos de Oliveira Rabelo Bassalo Coutinho^3^, João Rafael Assis Alderete^1^ , Júlia Chihondo Kanjongo^3^ , Renata Aguiar Menezes Silva^1^, Nathalia Sernizon Guimarães^4b^, Ana Cristina Simões e Silva^1b^, Vandack Nobre^4,5b^

*^a^ Both authors contributed equally to this article as first authors.*

*^b^ All authors contributed equally to this article as senior authors.*

*^1^ Interdisciplinary Laboratory of Medical Investigation (LIIM), School of Medicine, Universidade Federal de Minas Gerais (UFMG), Belo Horizonte, MG, Brazil*

*^2^ School of Medicine, Faculdade de Saúde e Ecologia Humana (FASEH, Vespasiano, MG, Brazil)*

*^3^ School of Medicine, Universidade Federal de Minas Gerais (UFMG), Belo Horizonte, MG, Brazil*

*^4^ Internal Medicine Department, School of Medicine, Universidade Federal de Minas Gerais (UFMG), Belo Horizonte, MG, Brazil*

*^5^ Núcleo Interdisciplinar de Investigação em Medicina Intensiva (NIIMI), Universidade Federal de Minas Gerais (UFMG), Belo Horizonte, MG, Brazil*

**Address correspondence to this author at the Dr. Vandack Nobre, Internal Medicine Department, School of Medicine, Universidade Federal de Minas Gerais (UFMG), Belo Horizonte, MG, Brazil, Avenida Professor Alfredo Balena, 110, Hospital das Clínicas, Unidade de Cuidados Intensivos do Adulto, Terceiro Andar, Ala Leste - Santa Efigênia, ZIP Code: 30130-100 Belo Horizonte, Brazil. E-mail: vandack@gmail.com*

**2023**

# POTENTIAL OF PUBLICATION BIAS


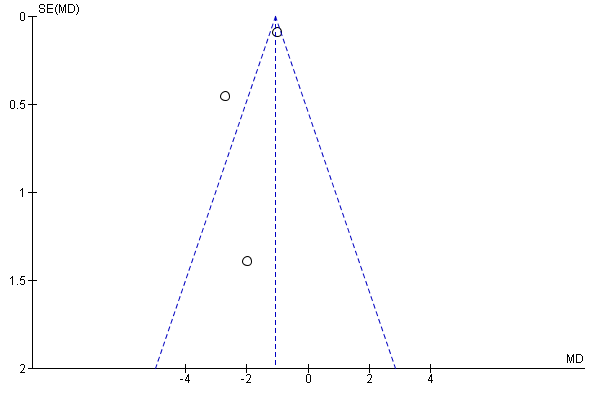


**Figure S1.** Funnel plot - Risk of bias of publication. Outcome: Duration of antibiotic therapy (days).


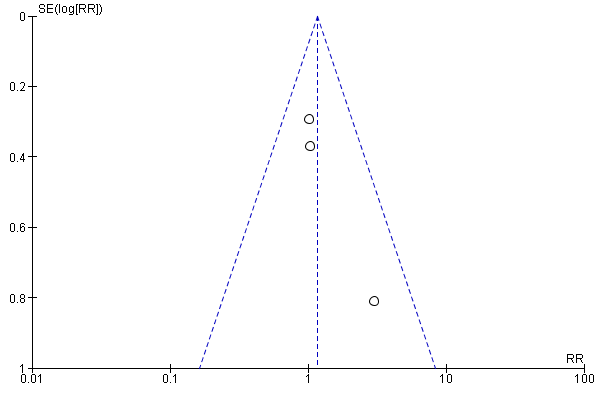


**Figure S2.** Funnel plot - Risk of bias of publication. Outcome: All-cause hospital mortality.


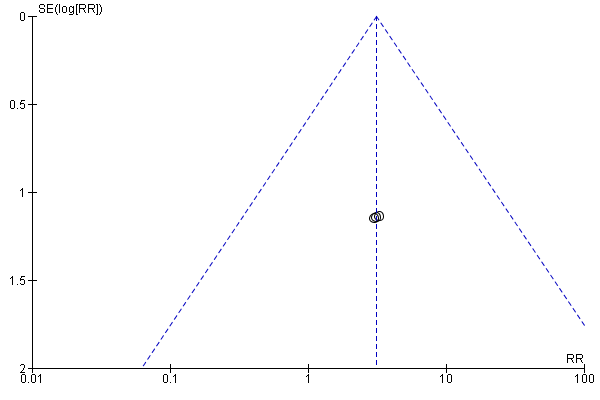


**Figure S3.** Funnel plot - Risk of bias of publication. Outcome: Relapse of infection.
